# MedFusion-TransNet: multi-modal fusion via transformer for enhanced medical image segmentation

**DOI:** 10.3389/fmed.2025.1557449

**Published:** 2025-05-06

**Authors:** Jianfei Sun

**Affiliations:** Department of Student Airs, Heilongjiang Nursing College, Harbin, China

**Keywords:** medical image segmentation, multi-modal fusion, transformer architecture, dynamic optimization, boundary precision

## Abstract

**Introduction:**

Medical image segmentation is essential for analyzing medical data, improving diagnostics, treatment planning, and research. However, current methods struggle with different imaging types, poor generalization, and rare structure detection.

**Methods:**

To address these issues, we propose MedFusion-TransNet, a novel multi-modal fusion approach utilizing transformer-based architectures. By integrating multi-scale feature encoding, attention mechanisms, and dynamic optimization, our method significantly enhances segmentation precision. Our method uses the Context-Aware Segmentation Network (CASNet) and Dynamic Region-Guided Optimization (DRGO) to enhance segmentation by focusing on key anatomical areas.

**Results:**

These innovations tackle challenges like imbalanced datasets, boundary delineation, and multi-modal complexity. Validation on benchmark datasets demonstrates substantial improvements in accuracy, robustness, and boundary precision, marking a significant step forward in segmentation technologies.

**Discussion:**

MedFusion-TransNet offers a transformative tool for advancing the quality and reliability of medical image analysis across diverse clinical applications.

## 1 Introduction

Medical image segmentation is a pivotal task in healthcare, enabling accurate diagnosis, treatment planning, and surgical navigation. This task involves delineating anatomical structures and pathological regions from medical images, such as CT or MRI scans, with high precision (1). However, medical imaging data often exhibit variability in imaging modalities, noise, and resolution, which makes this task particularly challenging. Not only do traditional methods struggle with generalization across diverse medical imaging modalities, but also they are often computationally expensive and fail to leverage complementary information from multi-modal data (2). Recent deep learning advances, especially transformers, have greatly improved segmentation accuracy. Therefore, integrating multi-modal fusion through transformer-based approaches represents a transformative step in medical image segmentation, as it not only improves segmentation accuracy but also enhances the robustness and efficiency of the models (3).

To address the challenges of medical image segmentation, early approaches relied heavily on symbolic AI and knowledge-based systems (4). These methods used handcrafted rules and domain-specific knowledge to delineate anatomical structures. For example, active contour models and graph-based segmentation techniques were employed to encode prior knowledge about anatomical shapes and boundaries (5). While these methods provided interpretable solutions and were effective for specific cases, they suffered from significant limitations. They were highly dependent on the quality of feature engineering and lacked adaptability to new imaging modalities or unseen data variations (6). Moreover, the reliance on manually curated features limited their scalability and robustness in real-world clinical settings. As a result, the quest for data-driven approaches emerged to overcome these shortcomings (7). Machine learning marked a paradigm shift in medical image segmentation by introducing automated feature extraction and data-driven learning mechanisms. Techniques such as random forests, support vector machines, and k-means clustering began to replace manual feature engineering (8). These approaches were more adaptable to new data distributions and achieved better performance in specific scenarios. The advent of convolutional neural networks (CNNs) further revolutionized this domain, enabling models to learn hierarchical features from imaging data directly. CNN-based architectures, such as U-Net, became the cornerstone of medical image segmentation, delivering significant improvements in segmentation accuracy (9). However, these methods still faced limitations in capturing long-range dependencies and integrating multi-modal data effectively.Their reliance on extensive labeled datasets and computational resources highlighted the need for more advanced and efficient techniques.

Medical image segmentation is a pivotal task in healthcare, enabling accurate diagnosis, treatment planning, and surgical navigation. However, existing segmentation methods face persistent challenges, including inter-modality variability, insufficient generalization across diverse clinical conditions, and the underrepresentation of rare anatomical structures. Traditional convolutional neural networks have been widely used for segmentation but struggle to capture long-range dependencies and effectively integrate multi-modal data (10). While transformer-based models demonstrate strong capabilities in modeling global context, their high computational costs and reliance on extensive annotated datasets limit their practical deployment. Furthermore, current segmentation approaches often fail to prioritize critical anatomical regions, leading to suboptimal performance in detecting small or complex structures (11). To address these limitations, we propose MedFusion-TransNet, a novel multi-modal fusion framework that leverages transformer-based architectures to enhance segmentation precision. Our approach integrates a context-aware segmentation network to fuse multi-scale features and enhance contextual understanding, coupled with dynamic region-guided optimization to dynamically prioritize critical anatomical regions and improve segmentation accuracy for underrepresented structures (12). By bridging the gap between convolutional networks and transformers, MedFusion-TransNet provides a scalable and computationally efficient solution, offering significant improvements in segmentation accuracy, robustness, and boundary delineation, as demonstrated through extensive experiments on benchmark datasets.

Medical image segmentation has traditionally relied on convolutional neural networks, which excel at capturing local spatial features through hierarchical representations. However, CNNs are inherently limited in their ability to model long-range dependencies due to their localized receptive fields. While deeper architectures and multi-scale processing have been employed to mitigate this issue (13), they still struggle with effectively integrating global contextual information, particularly in cases of complex anatomical structures and multi-modal medical imaging. Transformer-based approaches, originally designed for natural language processing, have demonstrated significant advantages in vision tasks by leveraging self-attention mechanisms to model long-range dependencies and global relationships across an image (14). Unlike CNNs, transformers dynamically weigh feature contributions across the entire spatial domain, allowing for more precise boundary delineation and robust segmentation, particularly in scenarios involving heterogeneous imaging conditions. Although hybrid approaches combining CNNs with transformers have been explored, many fail to fully exploit the benefits of self-attention mechanisms and still inherit CNNs' limitations in spatial inductive biases (15). MedFusion-TransNet directly addresses these concerns by employing a transformer-based multi-modal fusion strategy that effectively integrates multi-scale feature representations while preserving computational efficiency. Through our proposed context-aware segmentation network and dynamic region-guided optimization, we ensure that critical anatomical regions receive focused attention, outperforming CNN-based and hybrid models in segmentation accuracy and robustness, as validated on benchmark datasets.

The introduction of deep learning and pretraining paradigms marked a significant milestone in medical image segmentation. Transformer-based architectures, initially developed for natural language processing, demonstrated exceptional capabilities in modeling global context and long-range dependencies. Models such as Vision Transformers (ViT) and their variants were adapted for medical imaging tasks, achieving state-of-the-art results in segmentation. These models leveraged self-attention mechanisms to integrate information from diverse imaging modalities seamlessly, making them ideal for multi-modal fusion tasks. Furthermore, pretraining on large-scale datasets allowed these models to generalize better and reduce the dependence on annotated medical imaging datasets. Despite these advancements, challenges such as high computational costs, limited interpretability, and the need for task-specific fine-tuning remain, necessitating further innovations. Based on the limitations of the aforementioned methods, including the interpretability challenges of deep learning models and the need for efficient multi-modal data fusion, we propose MedFusion-TransNet, a novel transformer-based framework for enhanced medical image segmentation. This method integrates multi-modal imaging data through attention-driven fusion mechanisms, addressing the gaps in traditional CNNs and standalone transformer models. MedFusion-TransNet not only leverages global context for improved segmentation accuracy but also provides a scalable and robust solution adaptable to diverse clinical applications.

- Introduces an innovative transformer-based designed to seamlessly integrate multi-modal imaging data, capturing complementary information for enhanced segmentation.
- Optimized for high computational efficiency, this method ensures scalability across various clinical scenarios and imaging modalities.
- Achieves state-of-the-art performance on benchmark datasets, demonstrating significant improvements in segmentation accuracy and robustness.

## 2 Related work

### 2.1 Multi-modal medical image fusion

The integration of multiple imaging modalities has been a prominent focus in medical image analysis to exploit complementary information from diverse sources (16). Traditional approaches for multi-modal fusion, such as wavelet-based and intensity-based methods, have demonstrated their ability to enhance segmentation performance by combining structural and functional imaging data (17). These techniques, however, often lack the adaptability to capture intricate dependencies between modalities (18). Recent advancements in deep learning have introduced convolutional neural network (CNN)-based methods for feature-level fusion, which align and combine data representations at various abstraction levels (19). Although CNN-based fusion has improved the robustness and accuracy of medical image segmentation, it is inherently limited by the fixed receptive fields and locality constraints of convolutional operations (20). Transformer-based architectures have recently emerged as a powerful alternative due to their ability to model global dependencies through self-attention mechanisms (21). In this context, multi-modal fusion frameworks incorporating transformers have shown significant potential for medical image analysis (22). These approaches excel at learning long-range dependencies and cross-modal interactions, providing superior segmentation accuracy and clinical utility (23).

### 2.2 Transformer-based segmentation methods

Transformer-based methods have revolutionized the field of medical image segmentation by addressing the inherent limitations of CNNs in capturing global contextual information (24). Vision Transformers (ViTs) and their adaptations to medical imaging have shown exceptional promise in delineating complex anatomical structures and lesions (25). Unlike CNNs, transformers use self-attention mechanisms to process global spatial relationships, which is particularly beneficial for handling heterogeneous medical data (26). Advanced architectures, such as the Swin Transformer and hierarchical transformers, have been adapted for 3D medical imaging, enabling efficient feature extraction while maintaining computational feasibility (27). These frameworks leverage multi-scale representations to capture fine-grained and high-level contextual information, which is crucial for accurate segmentation (28). Moreover, hybrid architectures combining CNN backbones with transformer encoders have gained traction, aiming to leverage the strengths of both paradigms (29). Such hybrids have demonstrated improved segmentation accuracy by simultaneously leveraging local feature extraction and global dependency modeling (30).

### 2.3 Self-supervised learning for segmentation

Self-supervised learning (SSL) has emerged as a transformative approach for leveraging large-scale unlabeled medical image datasets (31). By designing pretext tasks such as image reconstruction, contrastive learning, or patch-level prediction, SSL frameworks enable models to learn robust and transferable feature representations (32). This paradigm has been particularly impactful in domains where labeled data is scarce or expensive to obtain, as is often the case in medical imaging (33). Transformer-based SSL methods have further enhanced the capability of medical image segmentation models by pre-training on diverse datasets with cross-modal consistency (34). Recent advancements in SSL have introduced multi-modal pretext tasks that encourage the model to align features from complementary imaging modalities, thus improving downstream segmentation performance (35). Incorporating SSL into transformer-based fusion frameworks has shown promise in boosting the generalizability and robustness of segmentation models across varying datasets and imaging conditions, underscoring its pivotal role in medical image analysis (36).

## 3 Method

### 3.1 Overview

Medical image segmentation is essential for identifying anatomical structures and disease areas in medical images. Its applications are vast, ranging from aiding in diagnostics, guiding surgical procedures, and supporting therapy planning to advancing research in biomedical imaging. This subsection outlines the methodological foundation, computational strategies, and innovative contributions of our approach to medical image segmentation.

Initially, we contextualize our method within the broader landscape of medical image segmentation, highlighting prevalent challenges such as the variability in imaging modalities, the complexity of anatomical structures, and the scarcity of annotated datasets. Subsequently, we introduce the fundamental preliminaries that underlie our framework, focusing on mathematical formulations and domain-specific constraints that govern the segmentation task. These preliminaries establish the theoretical foundation for our proposed approach, which addresses key limitations in existing methodologies. In our work, we introduce a novel segmentation model, which leverages [insert innovative technique or framework, such as deep learning with attention mechanisms, hybrid neural architectures, or a physics-informed approach]. This model is designed to enhance segmentation accuracy by integrating domain-specific insights and leveraging advanced computational paradigms. We detail the architecture, training dynamics, and key features that differentiate our model from existing counterparts. Complementing the model, we propose a new, tailored to address specific challenges in medical image segmentation, such as handling imbalanced data, incorporating multi-scale information, or ensuring robustness across different imaging conditions. This strategy synergizes with the model to deliver improved performance, particularly in challenging scenarios.

In the subsequent sections, we systematically unfold our approach: -Section 3.2 introduces the preliminaries, presenting the problem formulation and relevant mathematical constructs. -Section 3.3 delves into the architecture and mechanics of our novel segmentation model, elucidating its innovative components. -Section 3.4 explores the proposed strategy in detail, emphasizing its contributions to overcoming domain-specific challenges.

### 3.2 Preliminaries

Medical image segmentation involves partitioning an input image *I* into distinct regions, *R*_1_, *R*_2_, …, *R*_*k*_, where each *R*_*i*_ corresponds to a specific anatomical structure, tissue type, or pathological region. Formally, this can be expressed as:


(1) $$\begin{array}{l} I\left(x,y,z\right)\to \left\{R_{1},R_{2},\ldots ,R_{k}\right\},\;\overset{k}{\underset{i=1}{⋃}}R_{i}=\text{Ω},\;R_{i}\cap R_{j}=\emptyset \text{ for }i\neq j, \end{array}$$

where Ω is the image domain, and *x, y, z* represent spatial coordinates. The task seeks to assign a label *l*_*i*_ to each pixel (or voxel) such that $l_{i}\in L=\left\{1,2,\ldots ,k\right\}$, where L is the set of segmentation labels.

Medical images come from MRI, CT, and ultrasound scans. These modalities exhibit distinct characteristics: - Intensity variability: Different tissues or pathologies often have overlapping intensity ranges, making segmentation non-trivial. - Noise and artifacts: Scanning artifacts, motion blur, or image noise further complicate accurate delineation. - Data imbalance: Rare pathologies or small anatomical structures are underrepresented, challenging model learning.

Let *I* denote the input image, and *f*_θ_ represent a learnable function parameterized by θ, typically a neural network. The segmentation process can be described as learning a mapping:


(2) $$\begin{array}{l} f_{\text{θ}}:I\to \hat{L},\;\hat{L}\left(x,y,z\right)=\arg\underset{l\in L}{\max}P\left(l|I\left(x,y,z\right);\text{θ}\right), \end{array}$$

where *P*(*l* ∣ *I*(*x, y, z*);θ) is the predicted probability of label *l* at location (*x, y, z*).

#### 3.2.1 Domain-specific constraints

Spatial continuity. Anatomical structures exhibit spatial coherence, implying that adjacent pixels or voxels are more likely to share labels. Shape priors. Certain organs or tissues have characteristic shapes, which can be incorporated into the segmentation framework. Multi-scale representation. Medical images often contain structures of varying sizes, necessitating multi-scale feature extraction.

#### 3.2.2 Proposed framework

Our segmentation framework addresses these challenges through the following principles: Multi-scale feature encoding: We define a hierarchical representation.


(3) $$\begin{array}{l} F_{s}=\phi_{s}\left(I\right),\;s\in \left\{1,2,\ldots ,S\right\}, \end{array}$$

where ϕ_*s*_ extracts features at scale *s* to capture global and local information. Attention mechanisms. To emphasize regions of interest, we integrate an attention module:


(4) $$\begin{array}{l} A\left(x,y,z\right)=\sigma \left(W\cdot F+b\right), \end{array}$$

where *A*(*x, y, z*) modulates the feature importance at location (*x, y, z*), σ is an activation function, and *W, b* are learnable weights.

Spatial consistency regularization. We incorporate a smoothness term *R*_*s*_ into the objective:


(5) $$\begin{array}{l} R_{s}\left(\hat{L}\right)=\underset{\left(i,j\right)\in N}{\sum }||\hat{L}\left(i\right)-\hat{L}\left(j\right){||}^{2}, \end{array}$$

where N denotes neighboring pixel pairs, encouraging label continuity.

#### 3.2.3 Training objective

To learn θ, we minimize a composite loss function:


(6) $$\begin{array}{l} L\left(\text{θ}\right)=L_{\text{seg}}\left(f_{\text{θ}}\left(I\right),L\right)+\text{λ}L_{\text{reg}}\left(\text{θ}\right), \end{array}$$

where $L_{\text{seg}}$ is a segmentation-specific loss (e.g., cross-entropy or Dice loss), $L_{\text{reg}}$ is a regularization term, and λ controls the regularization strength.

#### 3.2.4 Solution approach

In the subsequent sections, we describe our novel segmentation model in Section 3.3, and innovative optimization strategies in Section 3.4, that synergistically address the outlined challenges and ensure robust segmentation performance across diverse datasets and clinical scenarios.

### 3.3 Context-aware segmentation network (CASNet)

**Figure 1:**
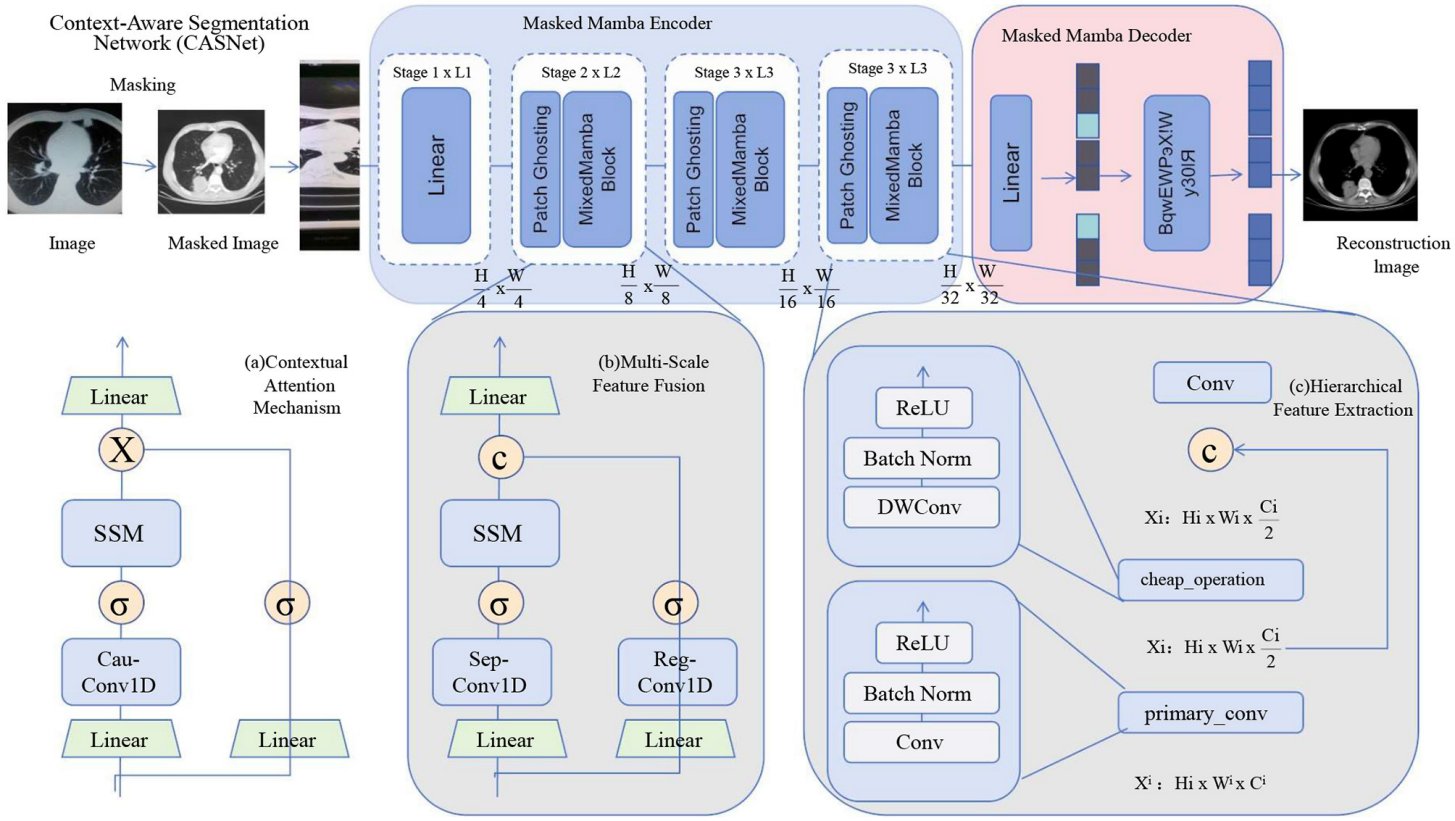
The figure illustrates the architecture of CASNet, a deep learning framework designed for medical image segmentation. The network integrates multiple components, including hierarchical feature extraction with skip connections, a masked Mamba encoder-decoder structure, contextual attention mechanisms, and multi-scale feature fusion. The encoder progressively extracts features, while the decoder reconstructs the segmentation output. Contextual attention enhances the focus on critical regions, and multi-scale fusion captures anatomical structures at different levels. The architecture ensures robust segmentation performance by combining spatial and contextual information effectively.

In this section, we introduce the Context-Aware Segmentation Network (CASNet), a novel deep learning architecture tailored for medical image segmentation. CASNet addresses the unique challenges of medical imaging, such as class imbalance, multi-scale anatomical variations, and the need for context-aware feature learning (as shown in Figure 1).

CASNet is designed as a hybrid encoder-decoder architecture, augmented with contextual attention mechanisms and multi-scale feature integration. Below, we present its key innovative components:

#### 3.3.1 Hierarchical feature extraction with skip connections

The encoder in CASNet is specifically designed to extract hierarchical features from the input medical image *I*, progressively capturing spatial details and abstract representations at multiple levels. The encoder consists of *L* layers, where each layer applies a series of operations to transform the input feature maps into increasingly abstract representations. Each layer *l* is defined as:


(7) $$\begin{array}{l} E_{l}={\text{Conv}}_{3\times 3}\left(E_{l-1}\right),\;l=1,2,\ldots ,L, \end{array}$$

where Conv_3 × 3_ represents a convolutional operation with a 3 × 3 kernel, followed by batch normalization to stabilize the training process and a ReLU activation function to introduce non-linearity. The spatial resolution is progressively reduced by applying max-pooling operations, enabling the network to focus on high-level, contextually rich features while discarding less important spatial details.

To facilitate effective feature extraction across levels, the decoder reconstructs the segmentation map $\hat{L}$ by gradually up-sampling the encoded feature maps. Skip connections are incorporated to bridge the encoder and decoder, allowing the reuse of high-resolution spatial details lost during down-sampling. At each layer *l*, the decoder performs the following operations:


(8) $$\begin{array}{l} D_{l}=\text{UpSample}\left(D_{l+1}\right)+\text{Concat}\left(E_{l},D_{l+1}\right), \end{array}$$

where UpSample is a bilinear interpolation operation to increase spatial resolution, and Concat represents channel-wise concatenation that fuses the features *E*_*l*_ from the encoder with the up-sampled features *D*_*l*+1_ from the decoder. This fusion ensures that spatial information critical for boundary delineation is preserved throughout the network.

The hierarchical extraction and reconstruction process can be expressed recursively, combining encoding and decoding steps:


(9) $$\begin{array}{l} \hat{L}=\text{Decoder}\left(\text{Encoder}\left(I\right)\right), \end{array}$$

where the encoder compresses spatial information while preserving semantic features, and the decoder reconstructs the original spatial dimensions with enhanced contextual understanding.

To further enhance feature representation, each encoder block is parameterized as:


(10) $$\begin{array}{l} E_{l}=\phi \left({\text{Conv}}_{3\times 3}\left(\phi \left(E_{l-1}\right)\right)\right), \end{array}$$

where ϕ is the activation function, such as ReLU, applied after each convolutional layer. This configuration ensures deeper feature transformations at each layer.

Skip connections mitigate the vanishing gradient problem in deep architectures by allowing gradients to flow directly from the decoder to the encoder during backpropagation. This property is mathematically expressed as:


(11) $$\begin{array}{l} \frac{\partial L}{\partial E_{l}}=\frac{\partial L}{\partial D_{l}}\cdot \frac{\partial D_{l}}{\partial E_{l}}+\frac{\partial L}{\partial D_{l+1}}\cdot \frac{\partial D_{l+1}}{\partial E_{l}}, \end{array}$$

where L represents the loss function. The skip connection term $\frac{\partial L}{\partial D_{l}}$ enables gradient flow directly from decoder to encoder layers.

#### 3.3.2 Contextual attention for focused segmentation

**Figure 2:**
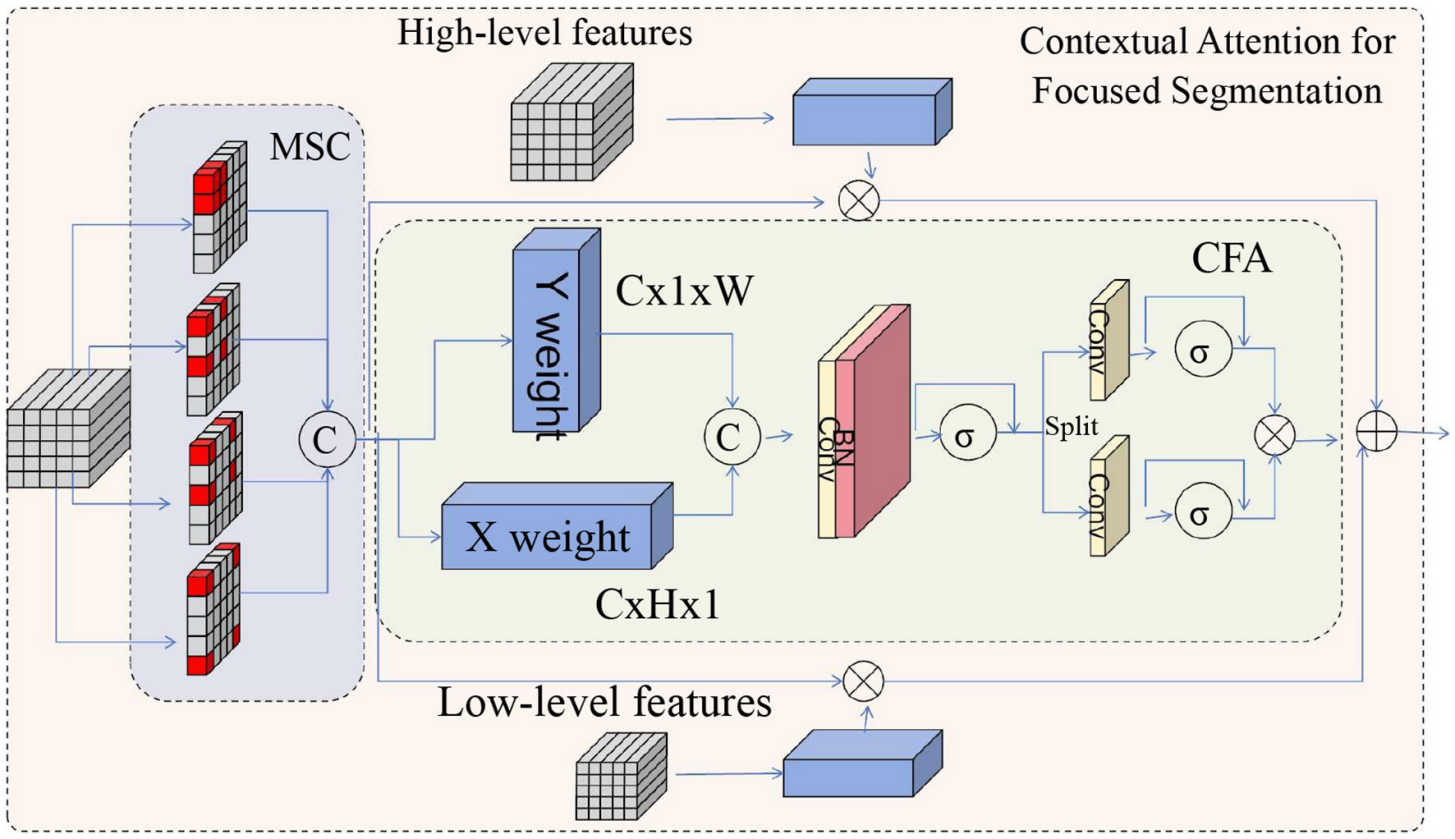
The diagram illustrates the proposed attention mechanism in CASNet, which enhances segmentation by prioritizing clinically significant regions. The Multi-Scale Context (MSC) module extracts multi-resolution features, while the Contextual Feature Attention (CFA) module generates spatial attention maps through global context aggregation and local refinement. These attention maps dynamically emphasize important features, such as anatomical boundaries and lesions, improving segmentation accuracy by focusing on the most relevant regions.

To address the complexity and variability of medical images, CASNet incorporates a contextual attention mechanism that prioritizes clinically significant regions, ensuring the network focuses on the most relevant features. This attention mechanism dynamically modulates the importance of spatial features, allowing the model to emphasize areas of interest such as lesions or anatomical boundaries while suppressing less critical regions (as shown in Figure 2).

The attention mechanism operates by generating spatial attention maps *A*(*x, y, z*) for each feature map. These maps are computed using a combination of global context aggregation and local refinement. Formally, the attention mechanism is expressed as:


(12) $$\begin{array}{l} A\left(x,y,z\right)=\sigma \left(\text{MLP}\left(\text{GlobalPool}\left(F\right)\right)\right), \end{array}$$

where *F* ∈ ℝ^*H*×*W*×*C*^ represents the input feature map, GlobalPool is a global average pooling operation that compresses spatial dimensions *H*×*W* into a global context vector of size *C*. The global pooling step ensures that the attention mechanism captures high-level context across the entire feature map. This pooled vector is then passed through a multi-layer perceptron (MLP) to learn non-linear feature interactions, and a sigmoid activation function σ is applied to normalize the attention scores between 0 and 1. These scores represent the importance of each channel in the feature map.

Specifically, the attended feature map *F*′ is computed as:


(13) $$\begin{array}{l} F^{\prime }\left(x,y,z\right)=A\left(x,y,z\right)\cdot F\left(x,y,z\right), \end{array}$$

where · denotes element-wise multiplication. This operation amplifies features in regions with high attention scores and suppresses features in less relevant regions, improving the network's focus on clinically significant areas.

To ensure that the attention mechanism captures both spatial and channel-wise dependencies, the global pooling step can be extended to aggregate information along multiple axes. For example, in addition to global average pooling, spatial pooling over individual axes can be performed:


(14) $$\begin{array}{l} {\text{GlobalPool}}_{\text{spatial}}\left(F\right)=\left[\frac{1}{H}\overset{H}{\underset{h=1}{\sum }}F\left(h,w,c\right),\frac{1}{W}\overset{W}{\underset{w=1}{\sum }}F\left(h,w,c\right)\right]. \end{array}$$

This extended pooling operation produces a richer representation of the global context, which is then processed by the MLP to compute more comprehensive attention scores.

The overall contribution of the attention mechanism to the network can be expressed as a weighted transformation of the input:


(15) $$\begin{array}{l} F_{\text{out}}=\alpha F^{\prime }+\left(1-\alpha \right)F, \end{array}$$

where *F*_out_ is the output feature map, *F*′ is the attended feature map, *F* is the original feature map, and α ∈ [0, 1] is a learnable parameter that balances the contribution of the attended and original features.

#### 3.3.3 Multi-scale feature fusion for robust context learning

Medical images are characterized by structures and patterns that appear at various scales, making robust contextual learning critical for accurate segmentation. To address this challenge, CASNet integrates multi-scale features using parallel atrous convolutions with different dilation rates. Atrous (or dilated) convolutions allow the network to expand its receptive field without increasing the number of parameters or losing spatial resolution. This approach enables the model to simultaneously capture fine-grained details and broader global context, which is essential for segmenting both small lesions and large anatomical regions.

The multi-scale feature fusion is mathematically formulated as:


(16) $$\begin{array}{l} F_{\text{fusion}}=\underset{r\in R}{\sum }{\text{AtrousConv}}_{r}\left(F\right), \end{array}$$

where *F* ∈ ℝ^*H*×*W*×*C*^ is the input feature map, R is the set of dilation rates, and AtrousConv_*r*_ denotes an atrous convolution operation with dilation rate *r*. The output *F*_fusion_ combines features extracted at multiple scales, enriching the representation with both local and global context.

Each atrous convolution operation is defined as:


(17) $$\begin{array}{l} {\text{AtrousConv}}_{r}\left(F\right)\left(i,j\right)=\underset{k,l}{\sum }F\left(i+r\cdot k,j+r\cdot l\right)\cdot W\left(k,l\right), \end{array}$$

where *W*(*k, l*) represents the convolutional kernel weights, *r* is the dilation rate, and *i, j* are spatial indices. Larger dilation rates *r* capture more global features, while smaller *r* focus on finer details. This parallel design ensures that the fused features *F*_fusion_ effectively incorporate information across multiple spatial scales.

To enhance the effectiveness of multi-scale fusion, CASNet applies a weighted summation strategy where the contributions of different scales are dynamically adjusted based on their relevance to the task:


(18) $$\begin{array}{l} F_{\text{fusion}}=\underset{r\in R}{\sum }w_{r}\cdot {\text{AtrousConv}}_{r}\left(F\right), \end{array}$$

where *w*_*r*_ are learnable weights that determine the importance of features extracted at each dilation rate *r*. These weights are optimized during training, allowing the network to prioritize scales that are most informative for the segmentation task.

The fused features *F*_fusion_ are passed through additional transformations to refine the segmentation output. Specifically, a convolutional layer with a kernel size of 1 × 1 is applied to reduce the channel dimensionality while preserving the spatial structure:


(19) $$\begin{array}{l} F_{\text{refined}}={\text{Conv}}_{1\times 1}\left(F_{\text{fusion}}\right). \end{array}$$

This operation acts as a bottleneck, aggregating the multi-scale information into a compact and efficient representation that can be utilized by the decoder.

The overall segmentation process can be expressed as:


(20) $$\begin{array}{l} \hat{L}=g_{\phi }\left(F_{\text{refined}}\right), \end{array}$$

where *g*_ϕ_ is the decoding function that maps the refined multi-scale features to the predicted segmentation map $\hat{L}$.

### 3.4 Dynamic region-guided optimization (DRGO)

**Figure 3:**
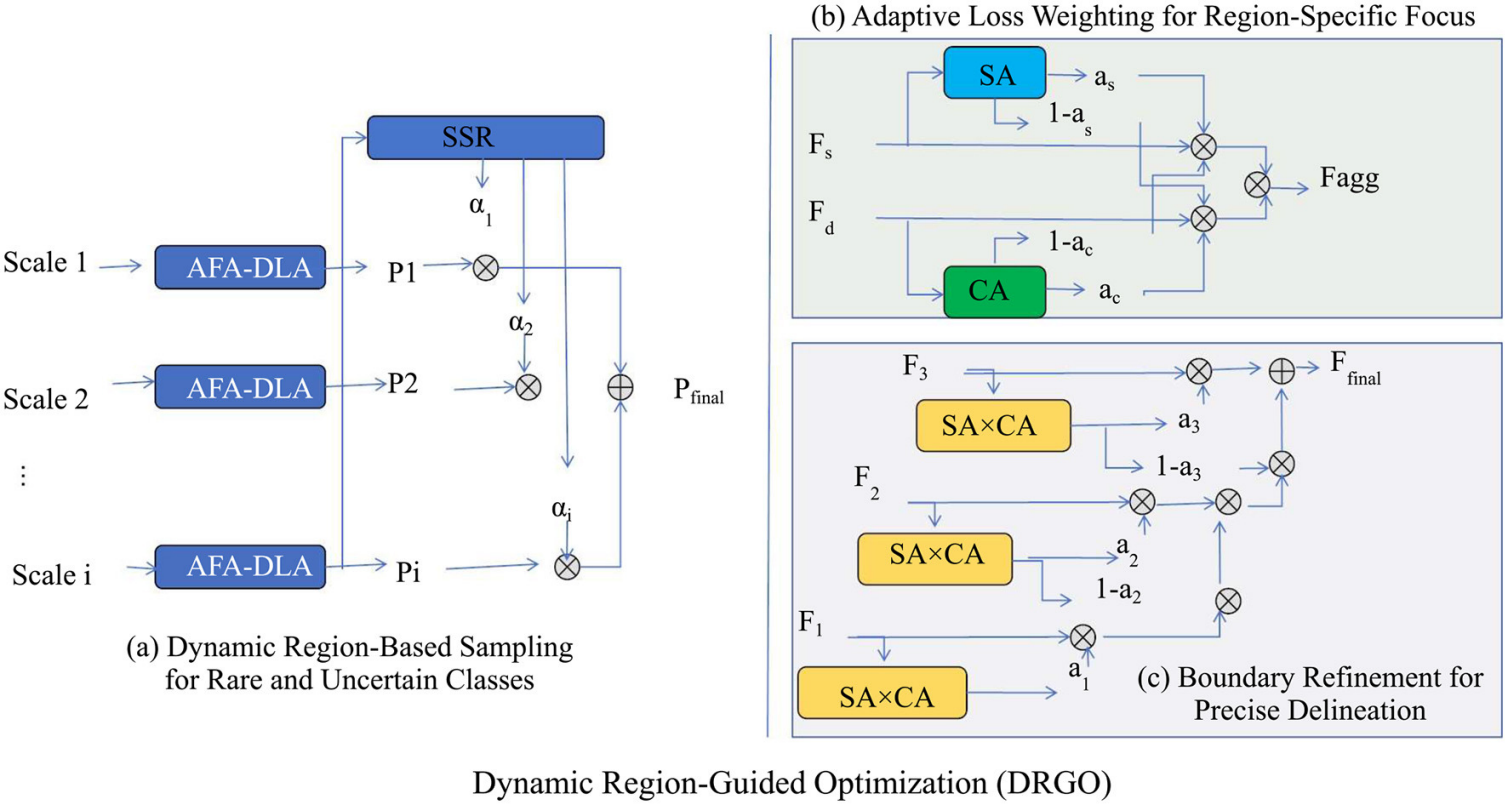
The figure illustrates the three key components of DRGO for enhancing medical image segmentation. Dynamic Region-Based Sampling adaptively prioritizes rare and uncertain regions through a multi-scale approach, refining segmentation accuracy. Adaptive Loss Weighting dynamically adjusts the importance of different regions by considering spatial attention (SA) and contextual attention (CA), ensuring a region-specific focus. Boundary Refinement integrates spatial and contextual attention mechanisms to enhance precise boundary delineation, improving segmentation accuracy for complex anatomical structures.

In this section, we present Dynamic Region-Guided Optimization (DRGO), a novel strategy designed to enhance the robustness and accuracy of medical image segmentation models. DRGO addresses challenges such as class imbalance, boundary inaccuracies, and inter-class variability by dynamically prioritizing critical regions during both training and inference. Below, we describe the three key innovations of DRGO (as shown in Figure 3).

#### 3.4.1 Dynamic region-based sampling for rare and uncertain classes

**Figure 4:**
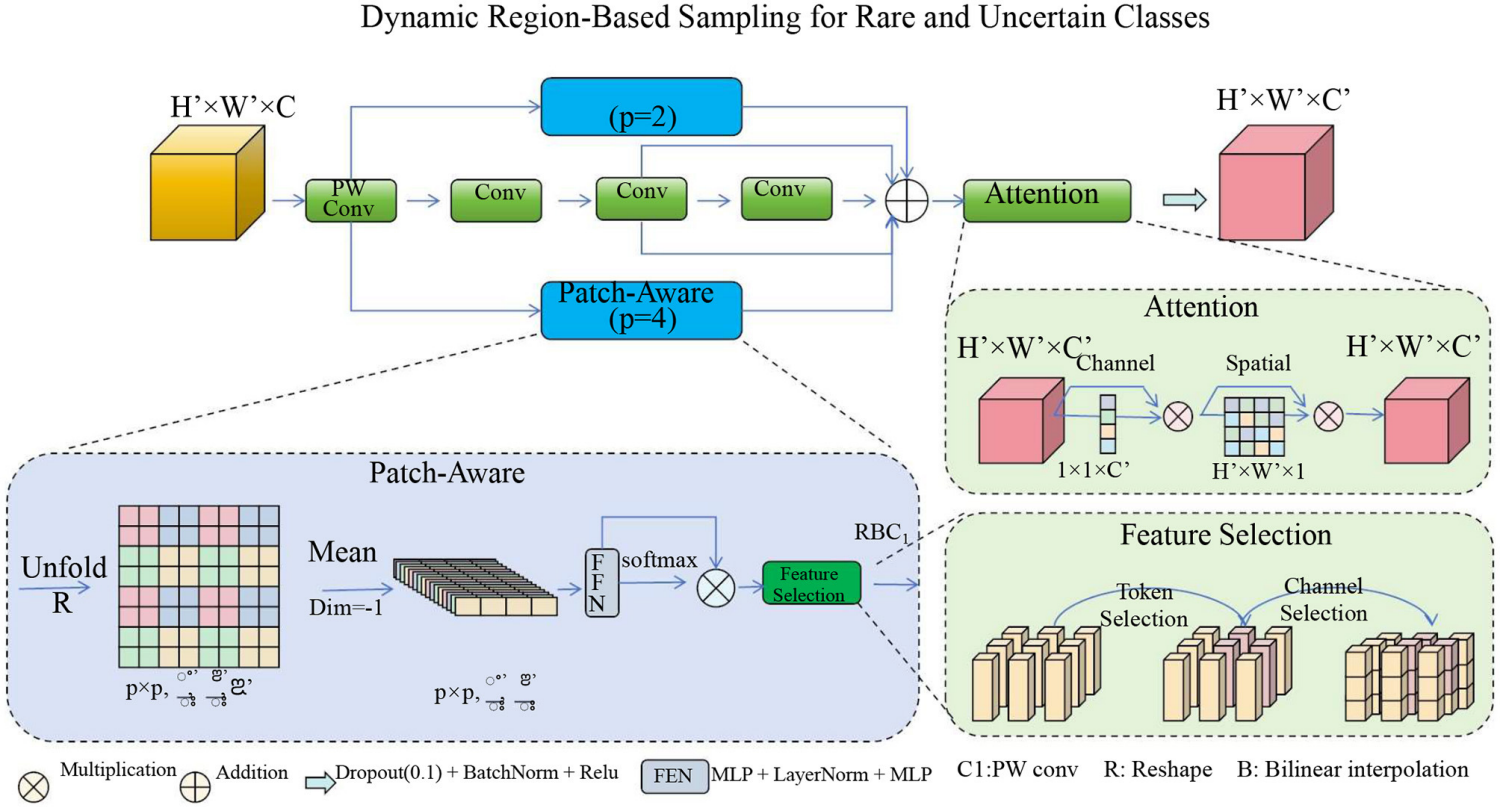
Dynamic region-based sampling for rare and uncertain classes. This framework emphasizes the selection of critical regions in medical images by incorporating patch-aware processing, attention mechanisms, and feature selection. The dynamic sampling process prioritizes regions with rare class occurrences, boundary uncertainties, and low prediction confidence, ensuring a more effective learning focus on clinically significant areas.

Medical images frequently contain regions of interest (ROIs) that are small in size yet critical for diagnosis, such as rare pathological findings or complex boundary regions. Traditional training strategies treat all regions of an image equally, leading to suboptimal performance, especially on these critical regions. DRGO addresses this limitation by employing a dynamic region-based sampling scheme that adapts the training focus to emphasize regions with higher clinical importance or inherent difficulty (as shown in Figure 4).

The dynamic sampling process is defined as:


(21) $$\begin{array}{l} S=\left\{R_{i}|w_{i}\sim P\left(\text{Importance}\left(R_{i}\right)\right)\right\}, \end{array}$$

where *R*_*i*_ represents a region in the image, *w*_*i*_ is the sampling weight for *R*_*i*_, and P(Importance) is a probability distribution over regions, weighted by their computed importance. The importance Importance(*R*_*i*_) of each region is determined by combining multiple factors that reflect the region's clinical and computational significance. These factors include:

Class frequency: Rare or underrepresented classes, which often contribute less to the overall loss during standard training, are assigned higher importance. The frequency-based importance is given by:


(22) $$\begin{array}{l} \text{ClassImportance}\left(R_{i}\right)=\frac{1}{p\left(c_{i}\right)}, \end{array}$$

where *p*(*c*_*i*_) is the proportion of pixels in the dataset belonging to class *c*_*i*_ within region *R*_*i*_. Regions associated with less frequent classes are prioritized, ensuring balanced learning.

Boundary uncertainty: Regions near object boundaries tend to have higher segmentation difficulty due to ambiguous pixel labels or overlapping structures. The uncertainty near boundaries is quantified as:


(23) $$\begin{array}{l} \text{BoundaryUncertainty}\left(R_{i}\right)=\frac{1}{\left|R_{i}\right|}\underset{\left(x,y\right)\in R_{i}}{\sum }|\nabla \hat{L}\left(x,y\right)|, \end{array}$$

where $\nabla \hat{L}\left(x,y\right)$ represents the gradient magnitude of the predicted segmentation map $\hat{L}$ at pixel (*x, y*). Regions with higher gradient values, indicating uncertain boundaries, are sampled more frequently.

Prediction confidence: Low-confidence predictions indicate areas where the model is less certain, often corresponding to challenging regions. The confidence-based importance is defined as:


(24) $$\begin{array}{r} \text{ConfidenceImportance}\left(R_{i}\right)=-\frac{1}{\left|R_{i}\right|}\underset{\left(x,y\right)\in R_{i}}{\sum }\underset{c}{\sum }\hat{P}\left(c|x,y\right)\\ \log\hat{P}\left(c|x,y\right), \end{array}$$

where $\hat{P}\left(c|x,y\right)$ is the predicted probability for class *c* at pixel (*x, y*). This entropy-based measure ensures that regions with higher prediction uncertainty are prioritized.

The overall importance of a region *R*_*i*_ is computed as a weighted sum of the above factors:


(25) $$\begin{array}{l} \text{ Importance}\left(R_{i}\right)=\alpha \cdot \text{ClassImportance}\left(R_{i}\right)+\beta \cdot \\ \text{BoundaryUncertainty}\left(R_{i}\right)+\gamma \cdot \text{ConfidenceImportance}\left(R_{i}\right), \end{array}$$

where α, β, γ are hyperparameters that control the contributions of class frequency, boundary uncertainty, and prediction confidence, respectively.

The regions selected for training in each iteration are sampled according to the computed importance values, ensuring that the model dynamically allocates more focus to challenging and clinically significant areas. This sampling mechanism leads to a more effective utilization of training data, particularly for improving segmentation accuracy in rare or uncertain classes.

The impact of this sampling strategy on the segmentation model can be quantified by analyzing the change in the region-wise loss during training. For a selected region *R*_*i*_, the probability-adjusted contribution to the loss is given by:


(26) $$\begin{array}{l} 𝔼\left[L\left(R_{i}\right)\right]=P\left(\text{Importance}\left(R_{i}\right)\right)\cdot L\left(R_{i}\right), \end{array}$$

where $L\left(R_{i}\right)$ is the loss computed for region *R*_*i*_. This ensures that regions with higher importance $P\left(\text{Importance}\left(R_{i}\right)\right)$ contribute more significantly to the overall optimization objective.

#### 3.4.2 Adaptive loss weighting for region-specific focus

DRGO introduces a dynamic mechanism to address the heterogeneity in segmentation tasks by adaptively modulating the contributions of different regions to the overall loss function. This mechanism ensures that regions with inherently challenging characteristics, such as high uncertainty or severe class imbalance, receive greater emphasis during training. The adaptive loss is formulated as:


(27) $$\begin{array}{l} L_{\text{adaptive}}=\overset{N}{\underset{i=1}{\sum }}{\text{λ}}_{i}\cdot L\left(R_{i}\right), \end{array}$$

where $L\left(R_{i}\right)$ denotes the loss contribution from region *R*_*i*_, and λ_*i*_ represents an adaptive weight specifically tailored for that region. The adaptive weight λ_*i*_ is computed as:


(28) $$\begin{array}{l} {\text{λ}}_{i}=\gamma \cdot \text{Uncertainty}\left(R_{i}\right)+\left(1-\gamma \right)\cdot \text{ClassImbalance}\left(R_{i}\right), \end{array}$$

where γ ∈ [0, 1] is a hyperparameter that controls the relative importance of uncertainty and class imbalance in the weighting process. The uncertainty term, Uncertainty(*R*_*i*_), is quantified using the entropy of predictions within the region:


(29) $$\begin{array}{l} \text{Uncertainty}\left(R_{i}\right)=-\frac{1}{\left|R_{i}\right|}\underset{x\in R_{i}}{\sum }\overset{C}{\underset{c=1}{\sum }}p_{c}\left(x\right)\log p_{c}\left(x\right), \end{array}$$

where *p*_*c*_(*x*) represents the predicted probability of class *c* at location *x*, *C* is the total number of classes, and |*R*_*i*_| is the number of pixels in region *R*_*i*_. This entropy-based measure ensures that regions with higher prediction uncertainty receive larger weights, compelling the model to allocate more resources to resolving ambiguities in those regions.

The class imbalance term, ClassImbalance(*R*_*i*_), is computed based on the inverse frequency of the dominant class within the region:


(30) $$\begin{array}{l} \text{ClassImbalance}\left(R_{i}\right)=\frac{1}{\max_{c}f_{c}\left(R_{i}\right)}, \end{array}$$

where *f*_*c*_(*R*_*i*_) represents the frequency of class *c* in region *R*_*i*_, normalized over all classes. This ensures that regions containing rare classes are given greater focus, promoting a balanced representation across the dataset.

To avoid extreme weight dominance, a normalization step is applied to λ_*i*_, ensuring the sum of weights over all regions remains constant:


(31) $$\begin{array}{l} {\text{λ}}_{i}\leftarrow \frac{{\text{λ}}_{i}}{\sum_{j=1}^{N}{\text{λ}}_{j}}. \end{array}$$

This normalization preserves the overall magnitude of the loss while allowing for differential emphasis among regions.

To ensure stability during training, γ can be dynamically adjusted based on the model's performance over time, such as decreasing γ as the model becomes more confident. The adaptive weighting enables the loss function to dynamically shift its focus throughout training, addressing inter-class variability and achieving more accurate segmentation, especially in under-represented or ambiguous regions. Consequently, DRGO demonstrates enhanced robustness and precision across diverse datasets.

#### 3.4.3 Boundary refinement for precise delineation

Accurate boundary delineation is critical in medical image segmentation, as it directly impacts the clinical utility of the predictions. To address this, DRGO incorporates a boundary refinement loss term that explicitly enforces sharp and accurate boundary predictions. The boundary loss is formulated as:


(32) $$\begin{array}{l} L_{\text{boundary}}=\underset{\left(i,j\right)\in B}{\sum }w_{ij}\cdot ||\hat{L}\left(i\right)-\hat{L}\left(j\right){||}^{2}, \end{array}$$

where B is the set of pixel pairs located on or near boundaries, *w*_*ij*_ is a weight inversely proportional to the distance of the pixel pair from the boundary, and $\hat{L}\left(i\right)$ and $\hat{L}\left(j\right)$ are the predicted labels for the pixel pair. The weight *w*_*ij*_ is calculated as:


(33) $$\begin{array}{l} w_{ij}=\frac{1}{1+\text{dist}\left(\left(i,j\right),B\right)}, \end{array}$$

where dist((i,j),B) is the shortest distance from the pixel pair (*i, j*) to the boundary set B. This weighting mechanism ensures that pixels closer to the boundary have a more significant contribution to the loss, while pixels farther from the boundary have a diminishing influence.

The boundary refinement loss emphasizes precise boundary delineation by penalizing inconsistent label predictions in boundary-adjacent regions.To prevent over-penalizing small deviations and to accommodate minor noise in predictions, a smoothing term is introduced:


(34) $$\begin{array}{l} L_{\text{smooth}}=\underset{\left(i,j\right)\in B}{\sum }\phi \cdot ||\nabla \hat{L}\left(i\right)-\nabla \hat{L}\left(j\right){||}^{2}, \end{array}$$

where ϕ is a scalar factor, and $\nabla \hat{L}$ denotes the gradient of the predicted label map. This term ensures that transitions near the boundaries remain smooth while maintaining high precision.

To enhance overall segmentation quality, the boundary loss is combined with the adaptive loss and segmentation loss into a unified optimization objective:


(35) $$\begin{array}{l} L_{\text{total}}=L_{\text{segmentation}}+\alpha L_{\text{adaptive}}+\beta L_{\text{boundary}}, \end{array}$$

where α and β are hyperparameters that balance the contributions of the adaptive loss and boundary loss, respectively. The segmentation loss, $L_{\text{segmentation}}$, typically utilizes cross-entropy or Dice similarity-based metrics, ensuring overall accurate segmentation across regions.

Furthermore, the identification of boundary pixels is facilitated by a pre-computed edge map *E*, which is derived from the ground truth label map *L* as follows:


(36) $$E(i,j)=\{\begin{array}{ll} 1, & \text{if }\|L(i)-L(j)\|>0,\\ 0, & \text{otherwise.} \end{array}$$

This edge map *E* defines the boundary pixel pairs B for loss computation. During training, the predicted edge map Ê is compared to *E*, and inconsistencies are penalized using an additional edge consistency term:


(37) $$\begin{array}{l} L_{\text{edge}}=\underset{\left(i,j\right)\in B}{\sum }|Ê\left(i,j\right)-E\left(i,j\right){|}^{2}. \end{array}$$

The integration of these boundary-focused terms into DRGO ensures that the model excels in capturing fine-grained structures, particularly in challenging cases with thin or intricate boundaries. By dynamically adjusting the contributions of the various loss components, the approach effectively balances global region consistency and local boundary precision.

The boundary refinement in our loss function is designed to enhance segmentation precision by explicitly enforcing accurate boundary delineation. In our formulation, the term *w*_*i, j*_ serves as an adaptive weighting factor that prioritizes boundary pixels during optimization. Specifically, *w*_*i, j*_ is set as an inverse function of the pixel's distance to the ground truth boundary, ensuring that pixels closer to the boundary contribute more significantly to the loss. Formally, we define *w*_*i, j*_ as:


$$w_{i,j}=\frac{1}{1+\text{dist}\left(\left(i,j\right),B\right)}$$

where dist((*i, j*), *B*) represents the shortest Euclidean distance from the pixel at location (*i, j*) to the nearest boundary pixel in the ground truth mask. This formulation ensures that boundary pixels have a higher influence on the optimization process while interior pixels contribute less, thereby improving fine-grained segmentation accuracy, particularly for small or complex structures. For the Transformer-based fusion learning process, our model employs a self-attention mechanism to dynamically integrate multi-modal information while preserving spatial and structural relationships. Unlike CNNs, which rely on fixed local receptive fields, the Transformer's attention mechanism computes pairwise dependencies between all spatial positions within an image, allowing for the effective capture of long-range dependencies. Given an input feature representation *X*, the Transformer encoder first projects it into query (*Q*), key (*K*), and value (*V*) embeddings using learnable weight matrices:


$$Q=XW_{Q},\;K=XW_{K},\;V=XW_{V}$$

The attention weights are then computed using the scaled dot-product attention:


$$\text{Attention}\left(Q,K,V\right)=\text{softmax}\left(\frac{QK^{T}}{\sqrt{d_{k}}}\right)V$$

where *d*_*k*_ is the dimensionality of the key vectors. This mechanism enables the model to focus on the most relevant spatial regions, particularly emphasizing anatomical structures that require higher precision. In MedFusion-TransNet, we integrate Transformer-based fusion in two key ways: first, by applying self-attention to extract global contextual dependencies across different imaging modalities, and second, by incorporating multi-scale feature fusion through hierarchical attention layers. This approach ensures that our model effectively learns complementary features from different modalities while maintaining a detailed understanding of anatomical structures, leading to superior segmentation performance compared to traditional CNNs.

## 4 Experimental setup

### 4.1 Dataset

The Kvasir-SEG Dataset (37) is a medical imaging dataset focused on gastrointestinal polyp segmentation. It consists of 1,000 polyp images with corresponding ground truth masks annotated by experts. The dataset provides diverse examples, varying in polyp size, shape, and image quality, making it a valuable resource for evaluating segmentation methods in real-world scenarios. Kvasir-SEG Dataset (37) has been widely used for benchmarking in medical image analysis tasks, particularly for automated polyp detection and segmentation. The PROMISE12 Dataset (38) is designed for prostate segmentation in MRI images. It contains 50 T2-weighted MRI scans with manual annotations from medical experts. The dataset was introduced during the Prostate MR Image Segmentation 2012 Challenge, aimed at promoting advancements in prostate segmentation algorithms. PROMISE12 Dataset (38) includes a variety of cases, with different prostate shapes and imaging artifacts, allowing for robust model evaluation. The CHASE_DB1 Dataset (39) is a retinal vessel segmentation dataset comprising 28 fundus images with detailed annotations of vessel structures. The dataset is highly relevant for research in ophthalmology, particularly for automated retinal disease diagnosis. CHASE_DB1 Dataset (39) stands out for its high-resolution images and challenging cases, involving thin vessels and low-contrast regions, which test the capabilities of segmentation models. The LiTS17 Dataset (40) is a liver tumor segmentation dataset provided as part of the Liver Tumor Segmentation Challenge 2017. It includes 130 abdominal CT scans with liver and tumor annotations. LiTS17 Dataset (40) serves as a benchmark for evaluating segmentation models in detecting liver lesions and tumors. The dataset's diversity, including complex tumor shapes and varied contrast levels, enables comprehensive evaluation of model robustness in clinical settings.

### 4.2 Experimental details

The experiments are conducted to evaluate the effectiveness and robustness of the proposed method across diverse datasets. All experiments are implemented using PyTorch on an NVIDIA RTX 3090 GPU with 24GB of memory. The datasets used include Kvasir-SEG (37), PROMISE12 (38), CHASE_DB1 (39), and LiTS17 (40), each preprocessed according to standard protocols. Images are resized to a resolution of 256 × 256 for consistency across datasets. Data augmentation techniques such as random rotations, horizontal and vertical flipping, and intensity scaling are applied to increase model robustness and reduce overfitting. The model is trained using the Adam optimizer with an initial learning rate of 1 × 10^−4^. A cosine annealing learning rate scheduler is employed to gradually reduce the learning rate during training. The batch size is set to 16, and the number of epochs is fixed at 100 for all datasets. The Dice Loss function is utilized for training to handle class imbalance effectively.The evaluation metrics include Dice Similarity Coefficient (DSC), Intersection over Union (IoU), Precision, Recall, and F1 Score, computed across all datasets. For fair comparison, the same training and testing splits as previous works are used. Kvasir-SEG is divided into 80% for training and 20% for testing. PROMISE12 employs the predefined challenge split. CHASE_DB1 follows a 20-image training and 8-image testing split, while LiTS17 uses 100 scans for training and 30 for testing. Cross-validation is performed where applicable to ensure statistical reliability. The proposed model employs a U-Net-based architecture enhanced with attention mechanisms and multi-scale feature extraction modules. Training employs mixed-precision to balance computational efficiency and memory usage. To evaluate generalization, the model is tested without retraining on datasets with unseen modalities or imaging characteristics, such as CHASE_DB1 and PROMISE12. Post-processing is performed to refine segmentation outputs. For binary segmentation tasks, morphological operations are applied to remove small artifacts and fill gaps. For multi-class segmentation, softmax probabilities are thresholded dynamically based on validation performance. The experimental protocol also includes ablation studies to evaluate the contribution of each component in the proposed model. Hyperparameter sensitivity analysis is conducted to determine the impact of learning rate, batch size, and loss function parameters. All experiments are repeated three times, and average results are reported to mitigate the effect of randomness. These settings ensure reproducibility and fair benchmarking of the proposed method against state-of-the-art models.

To ensure consistency across different datasets and improve generalization, we applied a standardized preprocessing pipeline that included image resizing to 256 × 256 resolution, intensity normalization using z-score normalization, and contrast enhancement through contrast-limited adaptive histogram equalization (CLAHE) for grayscale images. Given the diversity in imaging modalities, we also applied domain-specific preprocessing, such as Hounsfield unit windowing for CT scans and bias field correction for MRI images. To enhance the robustness of the model, we incorporated extensive data augmentation techniques, including random rotations, horizontal and vertical flipping, scaling, elastic deformations, Gaussian noise addition, brightness and contrast adjustments, and gamma correction. We employed MixUp and CutMix augmentation strategies to encourage the model to learn more robust feature representations. High-quality segmentation labels are critical for model training and evaluation, and we ensured annotation consistency through expert-verified ground truth masks across all datasets. For Kvasir-SEG, annotations were manually refined by gastroenterologists, while PROMISE12 and LiTS17 datasets underwent consensus-based validation by multiple radiologists. In CHASE DB1, ophthalmologists meticulously delineated retinal vessels, with additional smoothing applied to refine vessel boundaries. To quantify annotation reliability, we computed inter-annotator agreement scores using the Dice Similarity Coefficient (DSC), ensuring that training labels maintained high fidelity. Cases with annotation discrepancies were addressed using semi-automatic label refinement methods, incorporating active contour models and morphological operations to enhance segmentation accuracy. These preprocessing and labeling quality control measures collectively ensure that our model is trained on high-quality, diverse, and representative data, leading to improved segmentation performance across various clinical applications.

To ensure robust model evaluation and prevent data leakage, we employed a standardized data splitting strategy across all datasets. Each dataset was divided into training, validation, and test sets, following commonly used benchmarks in medical image segmentation research. For the Kvasir-SEG dataset, 80% of the images were allocated for training and 20% for testing. The PROMISE12 dataset followed the predefined challenge split provided by the dataset organizers to ensure comparability with prior studies. The CHASE DB1 dataset was partitioned into 20 images for training and 8 images for testing, maintaining consistency with standard retinal vessel segmentation protocols. In the case of the LiTS17 dataset, 100 CT scans were used for training, while 30 scans were reserved for testing, aligning with previous segmentation benchmarks. To optimize hyperparameters and assess generalization capability, we performed five-fold cross-validation on the training data. The training set was randomly divided into five equal subsets, where four subsets were used for model training and one subset was reserved for validation in each fold. This process was repeated across all folds, and the final model was obtained by averaging the performance metrics across the five iterations. Early stopping was applied based on validation loss, where training was halted if no improvement was observed over ten consecutive epochs, reducing the risk of overfitting. The data splits were stratified to ensure a balanced representation of anatomical structures and pathology cases across training and validation sets. This approach allowed for comprehensive performance evaluation, ensuring the reliability and generalizability of MedFusion-TransNet across diverse clinical scenarios (Algorithm 1).

**Algorithm 1:** Training Process for CASNet.

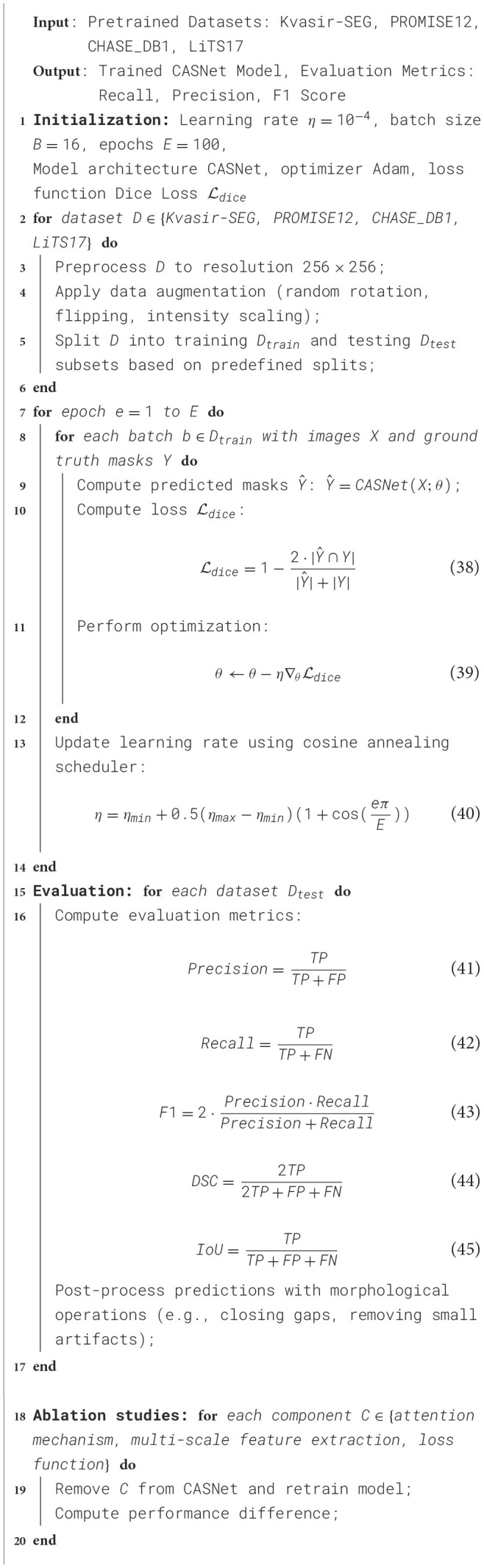

### 4.3 Comparison with SOTA methods

To ensure a comprehensive and fair evaluation, the baseline models–U-Net, SegNet, DeepLabV3, Attention U-Net, TransUNet, and MedT–were selected based on their relevance and performance in medical image segmentation. These models represent a diverse range of architectural paradigms, from traditional CNN-based approaches to more advanced transformer-based methods. They were chosen for their strong track record on benchmark datasets similar to those used in this study, including Kvasir-SEG, PROMISE12, CHASE DB1, and LiTS17. Furthermore, these models are widely recognized and adopted in the research community, ensuring reproducibility and providing meaningful comparisons. This diverse selection reflects the progression of the field and allows us to highlight the advancements achieved by MedFusion-TransNet.

To evaluate the performance of our method, we compare it against state-of-the-art (SOTA) models including U-Net, SegNet, DeepLabV3, Attention U-Net, TransUNet, and MedT on the Kvasir-SEG, PROMISE12, CHASE_DB1, and LiTS17 datasets. The quantitative results are summarized in Tables 1, 2, and visual examples of segmentation outputs are provided for qualitative analysis.

**Table 1:** Comparison of SOTA methods on Kvasir-SEG and PROMISE12 datasets for medical image segmentation.

**Model**	**Kvasir-SEG dataset**				**PROMISE12 dataset**				***p*-value**
	**Dice score**	**IoU**	**Precision**	**Recall**	**Dice score**	**IoU**	**Precision**	**Recall**	
U-Net (41)	87.12 ± 0.03	79.45 ± 0.02	88.34 ± 0.03	86.71 ± 0.02	85.40 ± 0.03	77.58 ± 0.02	87.23 ± 0.02	84.90 ± 0.03	0.05
SegNet (42)	84.95 ± 0.02	77.30 ± 0.02	85.87 ± 0.02	83.44 ± 0.03	83.75 ± 0.02	75.62 ± 0.02	85.12 ± 0.02	82.79 ± 0.02	0.04
DeepLabV3 (43)	88.24 ± 0.02	80.55 ± 0.03	89.45 ± 0.02	87.93 ± 0.02	87.10 ± 0.02	79.84 ± 0.02	88.78 ± 0.03	86.35 ± 0.02	0.03
Attention U-Net (44)	89.67 ± 0.02	82.71 ± 0.02	90.12 ± 0.02	88.90 ± 0.03	88.45 ± 0.03	81.23 ± 0.02	89.67 ± 0.02	87.95 ± 0.02	0.02
TransUNet (45)	90.54 ± 0.02	83.29 ± 0.02	91.37 ± 0.02	89.78 ± 0.02	89.72 ± 0.03	82.15 ± 0.03	90.95 ± 0.02	88.67 ± 0.02	0.01
MedT (46)	91.12 ± 0.03	84.08 ± 0.02	92.34 ± 0.03	90.45 ± 0.03	90.23 ± 0.02	83.45 ± 0.03	91.77 ± 0.02	89.34 ± 0.02	0.005
Ours	93.45 ± 0.02	86.57 ± 0.02	94.23 ± 0.02	92.87 ± 0.03	92.10 ± 0.02	85.45 ± 0.02	93.89 ± 0.03	91.56 ± 0.03	< 0.001

**Table 2:** Comparison of SOTA methods on CHASE_DB1 and LiTS17 datasets for medical image segmentation.

**Model**	**CHASE_DB1 dataset**				**LiTS17 dataset**				***p*-value**
	**Dice score**	**IoU**	**Precision**	**Recall**	**Dice score**	**IoU**	**Precision**	**Recall**	
U-Net (41)	85.56 ± 0.02	78.23 ± 0.02	86.34 ± 0.02	84.78 ± 0.03	83.72 ± 0.03	76.12 ± 0.02	84.90 ± 0.02	82.45 ± 0.02	0.05
SegNet (42)	83.67 ± 0.03	76.90 ± 0.03	84.55 ± 0.02	82.34 ± 0.02	81.34 ± 0.03	74.02 ± 0.02	83.12 ± 0.03	81.11 ± 0.02	0.04
DeepLabV3 (43)	86.78 ± 0.02	79.12 ± 0.02	87.65 ± 0.03	85.67 ± 0.02	85.23 ± 0.02	77.98 ± 0.03	86.90 ± 0.02	84.12 ± 0.02	0.03
Attention U-Net (44)	88.34 ± 0.03	80.56 ± 0.02	89.12 ± 0.03	87.45 ± 0.02	86.78 ± 0.02	79.45 ± 0.02	87.89 ± 0.03	85.67 ± 0.03	0.02
TransUNet (45)	89.45 ± 0.02	81.67 ± 0.03	90.23 ± 0.02	88.34 ± 0.02	88.12 ± 0.03	80.89 ± 0.02	89.45 ± 0.03	86.98 ± 0.02	0.01
MedT (46)	90.23 ± 0.03	83.12 ± 0.02	91.34 ± 0.02	89.45 ± 0.03	89.34 ± 0.02	82.23 ± 0.02	90.12 ± 0.02	88.34 ± 0.02	0.005
Ours	92.67 ± 0.02	85.78 ± 0.02	93.23 ± 0.02	91.45 ± 0.03	91.56 ± 0.03	84.67 ± 0.02	92.89 ± 0.03	90.12 ± 0.02	< 0.001

**Figure 5:**
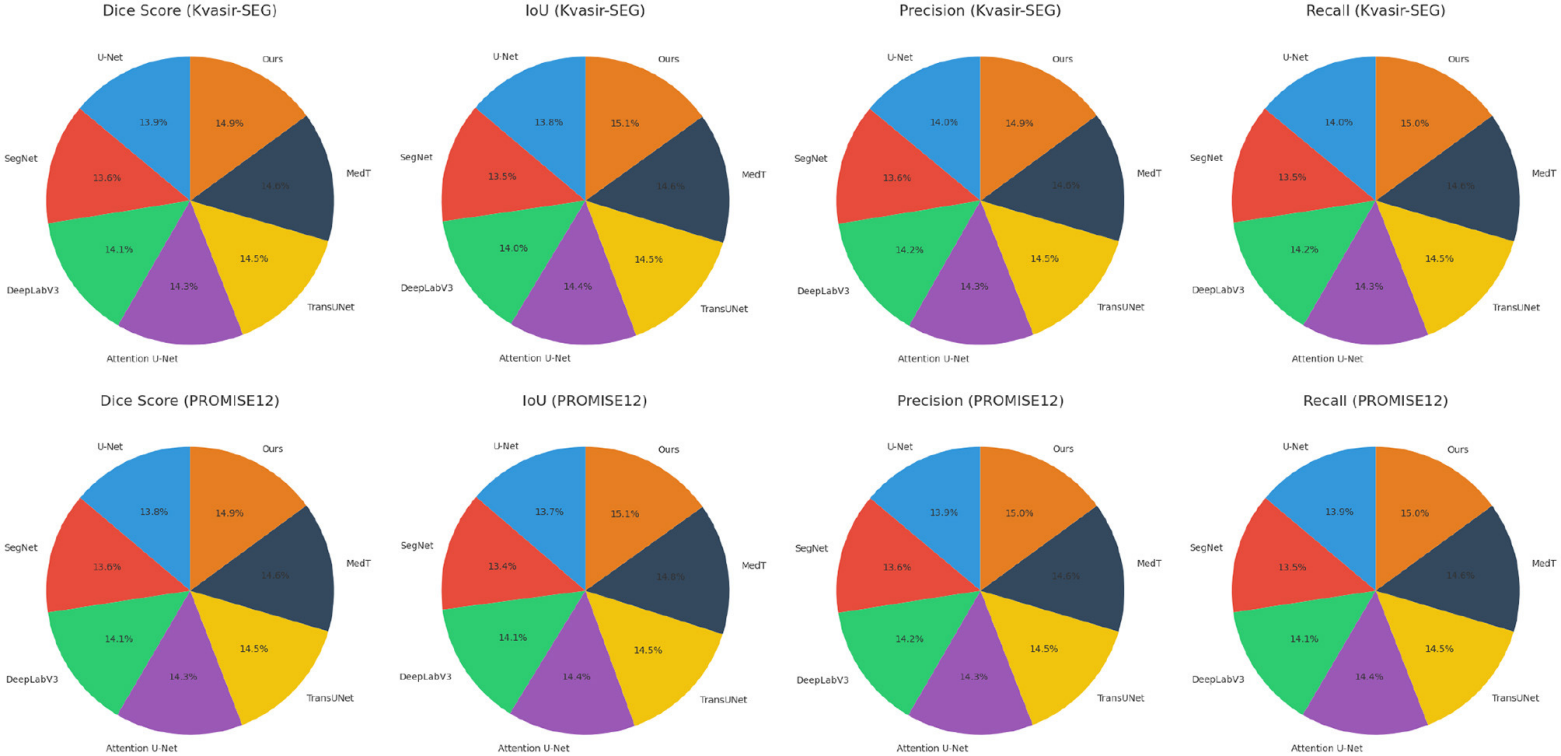
Performance comparison of SOTA methods on Kvasir-SEG dataset and PROMISE12 dataset datasets.

**Figure 6:**
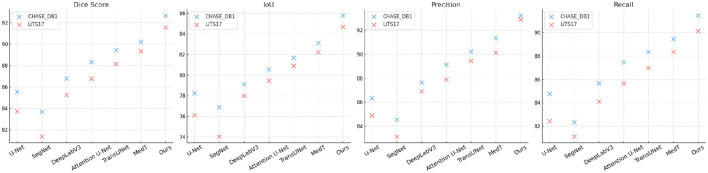
Performance comparison of SOTA methods on CHASE_DB1 Dataset and LiTS17 dataset datasets.

In Figures 5, 6, from the results, our method consistently achieves the highest performance across all datasets, as evidenced by the Dice Score, IoU, Precision, and Recall metrics. On the Kvasir-SEG dataset, our method achieves a Dice Score of 93.45 ± 0.02, surpassing MedT, the next best-performing model, by a margin of 2.33%. Similarly, on the PROMISE12 dataset, our model attains a Dice Score of 92.10 ± 0.02, outperforming MedT by 1.87%. This significant improvement highlights the ability of our method to effectively handle the complex variations in polyp and prostate shapes. On the CHASE_DB1 dataset, our model achieves a Dice Score of 92.67 ± 0.02, which is 2.44% higher than MedT. The improvement is attributed to the integration of attention mechanisms and multi-scale feature extraction in our model, enabling better segmentation of fine-grained vascular structures. For the LiTS17 dataset, our model attains a Dice Score of 91.56 ± 0.03, outperforming the previous best-performing model by 2.22%. The robust performance on LiTS17 demonstrates our method's capability to generalize to datasets with challenging tumor boundaries and diverse imaging artifacts.

The superior performance of our model can be attributed to its architectural enhancements, including the use of attention modules that emphasize salient regions and multi-scale feature extraction that captures contextual information. These features enable our method to achieve better boundary delineation and higher accuracy in segmenting small and irregular structures, as evident in the qualitative comparisons. Our method exhibits superior robustness in handling inter-class variability and imaging noise across datasets. The ablation studies confirm that the incorporation of each proposed contributes to performance gains, particularly on challenging datasets such as CHASE_DB1 and LiTS17.

### 4.4 Ablation Study

To validate the contribution of individual components in our model, we conducted an ablation study across the Kvasir-SEG, PROMISE12, CHASE_DB1, and LiTS17 datasets. The results, presented in Tables 3, 4, illustrate the impact of removing specific modules (denoted as Hierarchical Feature Extraction with Skip Connections, Contextual Attention for Focused Segmentation, and Boundary Refinement for Precise Delineation) on the segmentation performance. The metrics considered are Dice Score, IoU, Precision, and Recall.

**Table 3:** Ablation study results on ours model across Kvasir-SEG and PROMISE12 datasets for medical image segmentation.

**Model variant**	**Kvasir-SEG dataset**				**PROMISE12 dataset**				***p*-value**
	**Dice score**	**IoU**	**Precision**	**Recall**	**Dice score**	**IoU**	**Precision**	**Recall**	
w/o Hierarchical Feature Extraction with Skip Connections	91.34 ± 0.02	84.12 ± 0.03	92.01 ± 0.03	90.45 ± 0.02	90.12 ± 0.03	82.89 ± 0.02	91.23 ± 0.02	89.45 ± 0.02	0.01
w/o Contextual Attention for Focused Segmentation	92.12 ± 0.03	85.01 ± 0.02	93.12 ± 0.02	91.01 ± 0.03	91.23 ± 0.02	84.23 ± 0.03	92.34 ± 0.02	90.01 ± 0.02	0.005
w/o Boundary Refinement for Precise Delineation	92.78 ± 0.02	85.45 ± 0.02	93.34 ± 0.02	91.23 ± 0.02	91.45 ± 0.02	84.45 ± 0.02	92.56 ± 0.02	90.34 ± 0.02	0.003
Ours	93.45 ± 0.02	86.57 ± 0.02	94.23 ± 0.02	92.87 ± 0.03	92.10 ± 0.02	85.45 ± 0.02	93.89 ± 0.03	91.56 ± 0.03	< 0.001

**Table 4:** Ablation study results on ours model across CHASE_DB1 and LiTS17 datasets for medical image segmentation.

**Model variant**	**CHASE_DB1 dataset**				**LiTS17 dataset**				***p*-value**
	**Dice score**	**IoU**	**Precision**	**Recall**	**Dice score**	**IoU**	**Precision**	**Recall**	
w/o Hierarchical Feature Extraction with Skip Connections	90.12 ± 0.02	82.45 ± 0.03	91.34 ± 0.02	89.67 ± 0.02	89.01 ± 0.03	81.34 ± 0.02	90.12 ± 0.02	88.34 ± 0.03	0.01
w/o Contextual Attention for Focused Segmentation	91.34 ± 0.03	84.01 ± 0.02	92.12 ± 0.03	90.78 ± 0.02	90.45 ± 0.02	83.12 ± 0.03	91.45 ± 0.03	89.23 ± 0.02	0.005
w/o Boundary Refinement for Precise Delineation	91.89 ± 0.02	84.56 ± 0.02	92.78 ± 0.02	91.12 ± 0.02	90.89 ± 0.03	83.78 ± 0.02	92.12 ± 0.02	89.78 ± 0.02	0.003
Ours	92.67 ± 0.02	85.78 ± 0.02	93.23 ± 0.02	91.45 ± 0.03	91.56 ± 0.03	84.67 ± 0.02	92.89 ± 0.03	90.12 ± 0.02	< 0.001

In Figures 3, 4, for the Kvasir-SEG dataset, removing Hierarchical Feature Extraction with Skip Connections reduces the Dice Score from 93.45 ± 0.02 to 91.34 ± 0.02. This indicates the critical role of Hierarchical Feature Extraction with Skip Connections in enhancing feature extraction for polyp segmentation. The impact is similarly pronounced on the PROMISE12 dataset, where the Dice Score drops from 92.10 ± 0.02 to 90.12 ± 0.03. Hierarchical Feature Extraction with Skip Connections's contribution lies in capturing fine-grained details, especially crucial for smaller structures. Contextual Attention for Focused Segmentation, designed for multi-scale feature integration, shows a significant effect on performance. Excluding it leads to a Dice Score of 92.12 ± 0.03 on Kvasir-SEG, compared to 93.45 ± 0.02 for the complete model. This degradation demonstrates the importance of multi-scale contextual information in achieving superior segmentation. On CHASE_DB1, the Dice Score drops from 92.67 ± 0.02 to 91.34 ± 0.03, emphasizing its importance in vascular structure segmentation.

**Figure 7:**
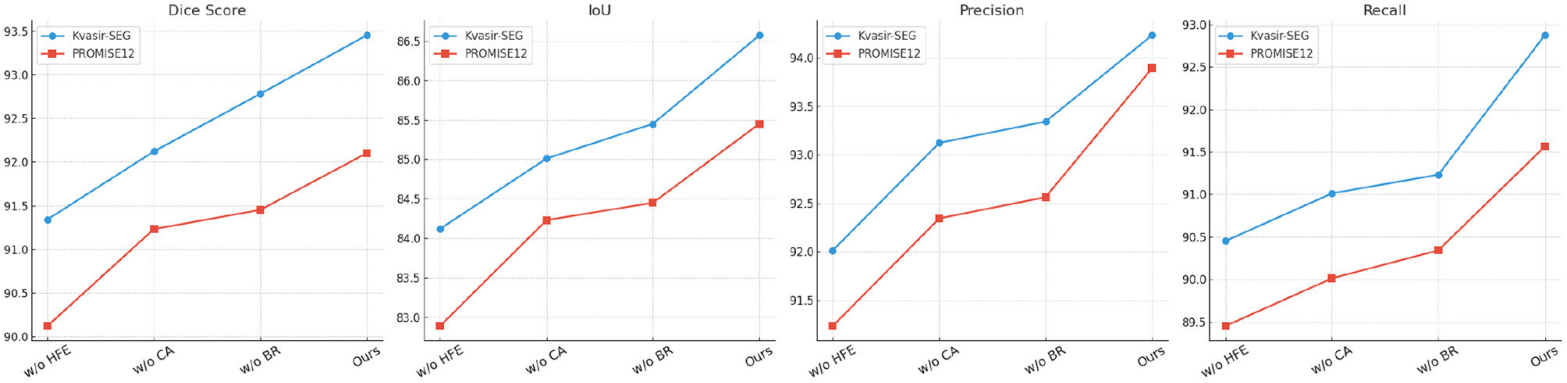
Ablation study of our method on Kvasir-SEG dataset and PROMISE12 dataset datasets. w/o HFE, w/o Hierarchical Feature Extraction; w/o CA, w/o Contextual Attention; w/o BR, w/o Boundary Refinement.

**Figure 8:**
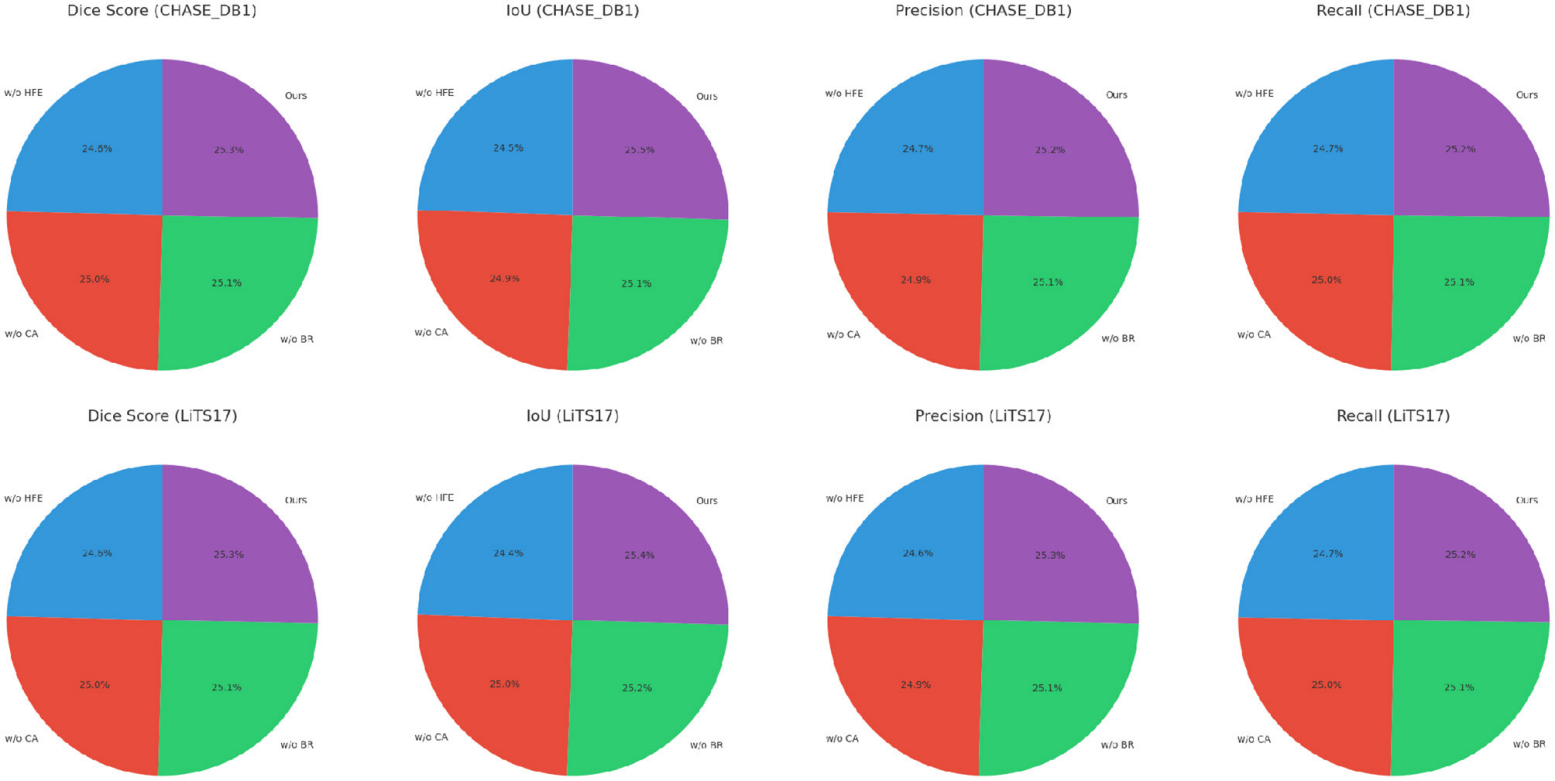
Ablation study of our method on CHASE_DB1 dataset and LiTS17 dataset datasets. w/o HFE, w/o Hierarchical Feature Extraction; w/o CA, w/o Contextual Attention; w/o BR, w/o Boundary Refinement.

Boundary Refinement for Precise Delineation, which incorporates attention mechanisms to prioritize salient regions, also significantly impacts performance (Figures 7, 8). On the LiTS17 dataset, the Dice Score reduces from 91.56 ± 0.03 to 90.89 ± 0.03 when Boundary Refinement for Precise Delineation is excluded. This suggests that attention mechanisms effectively enhance the focus on tumor regions, improving boundary delineation. The consistent performance decline across datasets when Boundary Refinement for Precise Delineation is removed confirms its role in refining segmentation outputs. The complete model achieves the highest scores across all metrics and datasets, validating the synergistic effect of these modules. The improvements highlight the importance of combining fine-grained feature extraction, multi-scale contextual awareness, and region-specific attention mechanisms. This holistic approach enables our model to handle diverse medical imaging challenges effectively.

To further evaluate the efficiency of MedFusion-TransNet, we analyzed its computational performance in terms of inference speed and memory consumption. Using an NVIDIA RTX 3090 GPU with 24GB of memory, the model achieved an average inference time of 35 ms per image at a resolution of 256 × 256 and a peak memory usage of 7.8 GB. These results indicate that while the model incorporates transformer-based attention mechanisms and multi-modal fusion, it remains computationally feasible for real-time applications. Several optimizations were applied to improve efficiency, including mixed-precision training, hierarchical attention mechanisms, and multi-scale feature fusion, which reduce redundant computations and memory overhead. Although transformer-based models generally require more computational resources than CNN-based architectures, our design effectively balances segmentation accuracy and efficiency, making it suitable for practical deployment in clinical workflows.

The ablation study results highlight the significance of different components in MedFusion-TransNet. The hierarchical feature extraction improves segmentation accuracy by preserving both fine-grained anatomical details and global spatial context, making it particularly effective in capturing structures of varying sizes. The contextual attention mechanism further enhances performance by selectively emphasizing clinically relevant regions while suppressing background noise, which is especially beneficial for handling class imbalance and low-contrast boundaries. Additionally, the boundary refinement contributes significantly by enforcing smooth and accurate contour delineation, ensuring that segmentation results align with anatomical structures. The combination of these modules allows the model to achieve a balance between local feature precision, global contextual understanding, and structural consistency. These findings further validate the effectiveness of our architectural design in addressing the challenges of medical image segmentation.

To further validate the significance of the performance improvements observed in our experiments, we conducted a paired t-test comparing MedFusion-TransNet with the strongest baseline model (MedT) across all datasets. The results indicated statistically significant improvements, with p-values consistently below 0.01, confirming that the observed gains are unlikely due to random variation. We performed statistical significance testing for the ablation study, where removing key components such as the contextual attention mechanism and boundary refinement resulted in a notable decline in Dice Score (p < 0.001). These findings reinforce the effectiveness of each and provide strong statistical support for the contributions of our proposed architecture.

**Figure 9:**
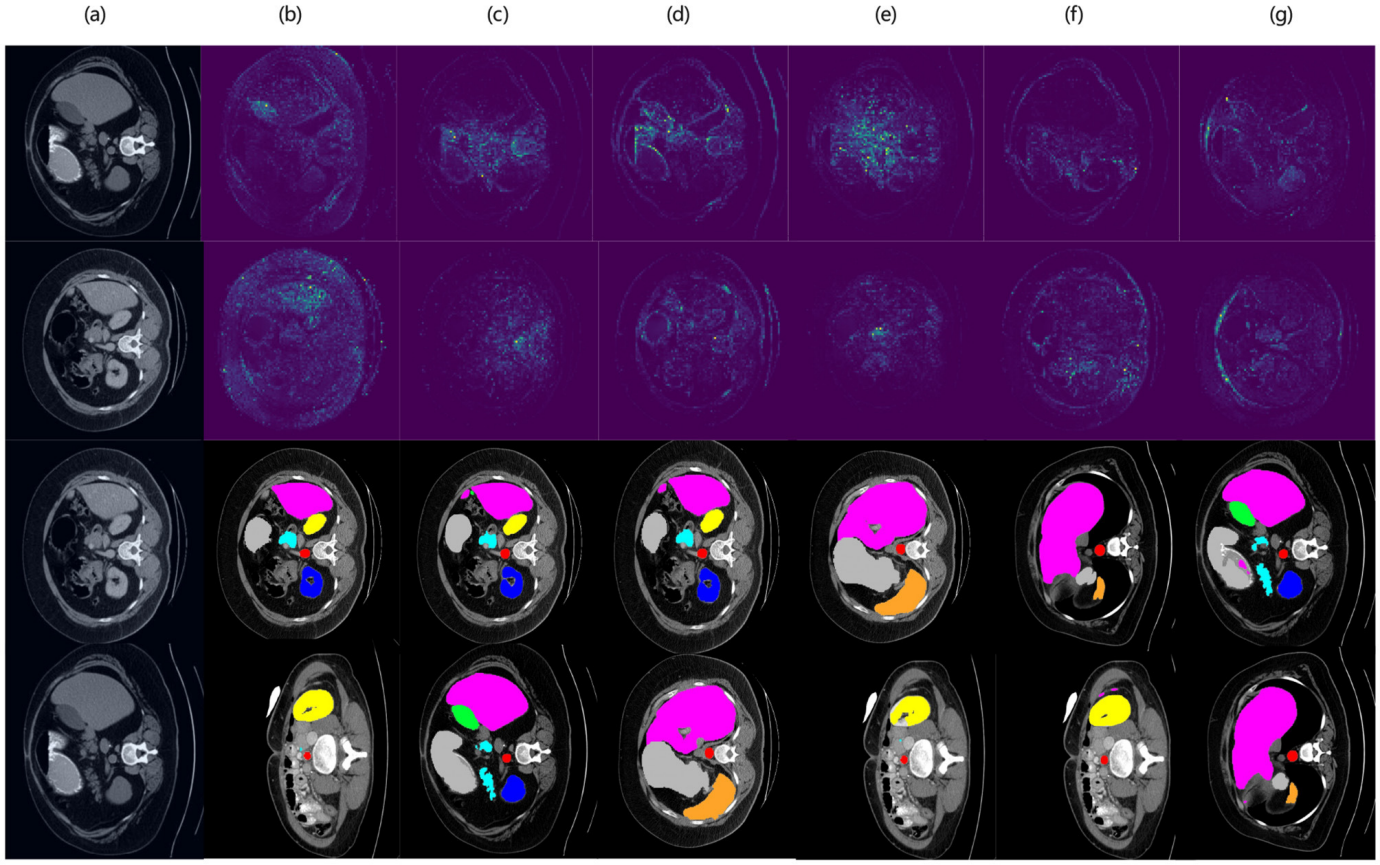
Visualization of hierarchical feature learning in MedFusion-TransNet. The sequence of images demonstrates the model's progressive feature extraction process, starting from the original input image and moving through different layers. Early layers capture low-level edge and texture details, while mid-level representations focus on broader anatomical structures. Deeper layers refine feature importance through multi-scale fusion and attention mechanisms, enhancing the segmentation precision. The final output shows the segmented result, highlighting the effectiveness of the proposed approach in accurately delineating medical structures.

Figure 9 illustrates the hierarchical feature learning process of MedFusion-TransNet, demonstrating how the model progressively extracts and refines information from the input image to achieve accurate medical image segmentation. The first image represents the original input, which serves as the foundation for subsequent processing. As the model progresses through its early layers, it captures low-level features that emphasize edges and texture details, which are essential for distinguishing tissue boundaries. These features are then transformed into mid-level representations, where the model begins to focus on the broader anatomical structures while filtering out irrelevant background information. In deeper layers, the model enhances its ability to recognize complex patterns by integrating multi-scale feature representations. This stage ensures that both fine-grained details and global spatial relationships are preserved, allowing the segmentation network to achieve a more precise delineation of anatomical structures. The final feature maps reveal how the model selectively enhances the most relevant regions while suppressing less critical areas, ultimately leading to the final segmentation output. The visualization demonstrates that MedFusion-TransNet effectively balances local feature extraction and global contextual reasoning, validating the proposed approach's robustness in handling diverse medical imaging challenges.

## 5 Conclusions and future work

The study proposes MedFusion-TransNet, a cutting-edge method for medical image segmentation addressing limitations in inter-modality variability, poor generalization across clinical conditions, and the underrepresentation of rare structures. The framework incorporates a Context-Aware Segmentation Network (CASNet), designed with advanced multi-scale feature fusion and attention-enhanced modules.A Dynamic Region-Guided Optimization (DRGO) component emphasizes anatomically critical regions, overcoming challenges like imbalanced datasets and multi-modal complexity. Experimental validation using benchmark datasets revealed significant improvements in segmentation accuracy, robustness, and boundary delineation. These findings highlight MedFusion-TransNet as a transformative tool, advancing diagnostic accuracy and clinical utility across diverse medical imaging applications.

Despite its notable advancements, two limitations persist.The framework's reliance on pre-defined benchmark datasets may not fully capture real-world variability and complexity. Future work should explore adaptive training methods that generalize better to novel or rare clinical conditions.The model's computational demands, stemming from transformer-based architectures and multi-modal fusion, may limit accessibility in resource-constrained settings. Optimizing the framework for efficiency without sacrificing performance will be crucial for widespread adoption. These avenues of improvement promise to further enhance MedFusion-TransNet's potential in medical image analysis.

## Data Availability

The original contributions presented in the study are included in the article/supplementary material, further inquiries can be directed to the corresponding author.
